# A Health Threat to Bystanders Living in the Homes of Smokers: How Smoke Toxins Deposited on Surfaces Can Cause Insulin Resistance

**DOI:** 10.1371/journal.pone.0149510

**Published:** 2016-03-02

**Authors:** Neema Adhami, Shelley R. Starck, Cristina Flores, Manuela Martins Green

**Affiliations:** 1 Department of Cell Biology and Neuroscience, University of California Riverside, Riverside, California, United States of America; 2 Department of bioshemistry and Byophysics/ Howard Hughes Medical Institute, Universty of California San Francisco, San Francisco, California, United States of America; The Ohio State University, UNITED STATES

## Abstract

Thirdhand smoke (THS) is the accumulation of secondhand smoke on environmental surfaces. THS is found on the clothing and hair of smokers as well as on surfaces in homes and cars of smokers. Exposure occurs by ingestion, inhalation and dermal absorption. Children living in homes of smokers are at highest risk because they crawl on the floor, touch parents’ clothing/hair and household objects. Using mice exposed to THS under conditions that mimic exposure of humans, we show that THS increases cellular oxidative stress by increasing superoxide dismutase (SOD) activity and hydrogen peroxide (H_2_O_2_) levels while reducing the activity of antioxidant enzymes catalase and glutathione peroxidase (GPx) that break down H_2_O_2_ into H_2_O and O_2_. This results in lipid peroxidation, protein nitrosylation and DNA damage. Consequences of these cell and molecular changes are hyperglycemia and insulinemia. Indeed, we found reduced levels of insulin receptor, PI3K, AKT, all important molecules in insulin signaling and glucose uptake by cells. To determine whether these effects on THS-induced insulin resistance are due to increase in oxidative stress, we treated mice exposed to THS with the antioxidants N-acetyl cysteine (NAC) and alpha-tocopherol (alpha-toc) and showed that the oxidative stress, the molecular damage, and the insulin resistance, were significantly reversed. Conversely, feeding the mice with chow that mimics “western diet”, which is known to increase oxidative stress, while exposing the mice to THS, further increased the oxidative stress and aggravated hyperglycemia and insulinemia. In conclusion, THS exposure results in insulin resistance in the form of non-obese type II diabetes (NODII) through oxidative stress. If confirmed in humans, these studies could have a major impact on how people view exposure to environmental tobacco toxins, in particular to children, elderly and workers in environments where tobacco smoke has taken place.

## Introduction

The adverse effects of Second-hand Smoke (SHS) are well known and documented, however the cellular and molecular consequences of exposure to Third-hand cigarette Smoke (THS) remain to be fully elucidated. THS consists of tobacco smoke toxins that linger on surfaces and in dust after tobacco has been smoked, including toxins that become increasingly toxic with age and are re-emitted into the air or react with other chemicals in the environment to yield new pollutants, including carcinogens. The aging and the multiple levels of exposure, ingestion, skin absorbption and inhalation, make THS a serious problem. It has been shown that this form of tobacco smoke remains in houses, apartments and hotel rooms after smokers move out [1,2] yet very little is known about the health effects of exposure to THS. In the US alone, nearly 88 million nonsmokers ages 3 and older live in homes where they are exposed to sufficient levels of SHS+THS to produce significant blood levels of cotinine (a metabolite of nicotine) and tobacco-specific nitrosamine, carcinogens that result from the reaction of nicotine with nitrous acid in the environment [3]. These metabolites have been found to be present in the urine of infants and children living in the homes of smokers [4–6]. In a separate study, an association was found between on-set of insulin resistance and type 2 diabetes in adolescents that grew up in households where at least one parent smoked cigarettes [7–10]. Alterations to the skeletal muscle mass and body fat composition have also been found to be associated with children living in the house of smokers, when compared to their counterparts not living in homes where smoking took place [11].

Cigarette smoking in general, has been associated with inflammation, oxidative stress, increased deposition of fat in the liver, alterations to the mitochondria and glucose metabolism pathways, as well as hyperglycemia and increased A1c levels, delineating the direct roles of these toxins in the onset of metabolic syndrome and insulin resistance [12–15]. There is mounting evidence regarding the generalized potential risks attributed to environmental cigarette smoke exposure, however, very little is known about the specific health implications of exposure to THS. It is thus critical to perform well-controlled experiments using animals, in order to evaluate the biological effects of THS-exposure that will then serve as a catalyst for subsequent human epidemiological experiments and clinical trials. Such studies can contribute to determining human health risks, design of clinical trials and potentially also contribute to development of policies that lead to reducing both exposure and disease.

To address this vital need, we have previously shown with an animal system using mice under conditions that mimic exposure of humans, that THS affects the physiology of several organ systems [16]. These mice are never exposed directly to SHS; the cages and the materials inside the cages are exposed to SHS and then animals placed in them. With this system we showed that significant damage occurs in liver, lung and during healing of wounds. In addition, the mice become hyperactive [16]. In the same study we presented data on the inability of the exposed mice to metabolize lipids and as a result have dyslipidemia and accumulation of lipids in the liver, much like in humans. The work presented here focuses on determining how THS exposure causes insulin resistance in the absence of obesity and how that affects skeletal muscle function.

## Materials and Methods

### Animals

C57BL/6 mice were divided into control and experimental groups. For exposure, mice were picked randomly. However, after exposure only mice that developed high blood glucose levels were used for the assays. By focusing on the affected population we should be able to obtain statistically significant data on the mechanisms of THS-induced insulin resistance. The experimental group was exposed to THS from weaning (three weeks of age) to 24 weeks without exposure to SHS at anytime during the study. The cages and the material inside were the only items exposed to the SHS. This smoke then lands and accumulates on the materials in the cage and thus is now THS. The control group was never exposed to THS. Mice were either fed a standard chow diet (percent calories: 58% carbohydrate, 28.5% protein, and 13.5% fat) or a “western diet” which is a modified chow diet similar to the high fat diet eaten by humans (percent calories 20% protein, 40% carbohydrates and 40% fat).

### Ethics Statement

All animal experimental protocols were approved by the University of California, Riverside, Institutional Animal Care and Use Committee (IACUC). Mice were euthanized with Carbon dioxide (CO_2_) inhalation which is the most common method of euthanasia used by NIH for mice. The levels and time of CO_2_ exposure were approved by the University of California, Riverside IACUC; death was induced quickly and without pain. The animal use protocol is A-2008024.

### THS Exposure

We used a Teague smoking apparatus [17] to generate and expose common household fabrics, placed in mouse cages to SHS smoke. Each cage contained 10g each of curtain material (cotton) and upholstery (cotton and fiber) and two 16 in^2^ pieces of carpet (fiber). In this manner we maintained equal exposure levels across experimental groups. Two packs of 3R4F research cigarettes were smoked each day, 5days/week and smoke was routed to a mixing compartment and distributed between two exposure chambers, each containing 4 cages with the materials.

We use a gravimetric method to determine the total particulate matter (TPM) concentrations. TPM refers to the sum of all the solid particles suspended in air and on surfaces in an enclosed space as a result of cigarette smoke (3–5). Whatman grade 40 quantitative cellulose filter papers are first weighed, then introduced into the filtering device on the vacuum, after which the suction is turned on for 15 mins. At the end of this period each filter is weighed again to determine the particulate mass that has accumulated during this time. This procedure is repeated with 2 more filters and the average of the 3 weights gives the TPM values for each chamber. The TPM is adjusted in our machine to match TPM levels found in the homes of smokers by the Environmental Protection Agency (EPA) by adjusting the flow rate of the smoke machine to 0.8liters/min. All cigarettes were smoked and stored in accordance with the Federal Trade Commission (FTC) smoking regimen [2].

At the end of each week, cages were removed from the exposure chamber, placed in plastic bags, and transported to the vivarium and the mice placed into the cages (S1 Fig). For the next week, an identical set of cages and fabric was then prepared and exposed to smoke in the same way as described above. By using two sets of cages and material, each of which were exposed on alternating weeks, we ensured that mice inhabited cages containing fabric that had been exposed (according to the regimen described above) to both fresh and aged THS. Throughout the exposure period, hair was removed weekly from the backs of the mice to mimic the bare skin of humans.

### Tissue Extracts

Frozen tissues were extracted to obtain total protein content. Quadriceps muscle was used, as it is the largest muscle with the most metabolic needs. Muscle tissue was homogenized in radioimmunoprecipitation assay buffer (RIPA), centrifuged, and supernatant collected. Extracts were used for immunoblots and oxidative stress assays. Glyceraldehyde phospahte dehydrogenase (GAPDH), a house keeping protein, was used as loading control. Bio-rad gel image software was used for densitometry and controlled exponential exposure (see S1 Methods).

### Western Blot analysis

We used procedure previously published by us [18] For detail see the S1 Methods.

### Oxidative Stress Determination

Hydrogen Peroxide will be determined using Caymans’s Chemical Hydrogen peroxide assay kit Cat# (7014200). SOD activity will be assayed using Cayman’s Chemical SOD activity kit (Cat# 706002). Catalase activity will be assayed using Cayman’s Chemical Catalase activity kit (Cat# 707002). GPx activity will be assayed using Cayman’s Chemical GPx activity kit (Cat# 703102). NADP/NADPH ratio: NADP/NADP will be determined using a kit from Biovision (Cat# K347). DNA Damage: we will use (8-OH-dG) ELISA assay kit from Cell BioLabs Inc (Cat# STA-320). Nitrosylation of protein: we will use OxiSelect Nitrotyrosine ELISA kit from Cell Biolabs Inc (Cat#STA-305). Lipid peroxidation: we will use OxiSelect TBARS assay kit from Cell Biolabs (Cat # STA-330). For all of these assays see S1 Methods.

### Blood Extraction

Blood was collected directly from the heart because it is the most effective way to collect a sufficient amount of sample for the various assays performed in this study. The blood was allowed to coagulate for 20-30mins, and then was subjected to centrifugation at 10,000 rpm to obtain phase separation. The upper phase was collected as serum and used immediately for assays that required fresh sample or frozen and stored at -80^°^C for later use.

### Blood Glucose Analysis

Fasting blood glucose levels were measured using a commercially available kit (TRUEresult) and gold sensor-laser accuracy strips. Mice were fasted for 12hrs in order to ensure that both glucose and insulin are at base levels. Both parameters are affected by food and water and that effect varies from animal to animal. Therefore, in order to make sure that we can compare the levels of glucose and of insulin amongst animals we need to fast them. To obtain fasting blood glucose levels, a nick was made on the tail vein and the glucose levels were measured by bringing the strip into contact with the drop of blood from the nicked tail vein.

### Fasting Serum Insulin concentration

We used the ALPCO Mouse Insulin ELISA kit (Cat# 80-INSMS-E01) and followed the protocol provided by the manufacturer. The plates were read at λ 450nm. Concentration of the samples was obtained from the standard curve.

### HOMA-IR

Homeostatic model assessment equation (HOMA) is a method for assessing insulin resistance (IR) derived by (fasting blood glucose X Fasting insulin)/22.5).

### Statistical Analysis

For the statistical analysis of experiments, we used Graphpad Instat Software (Graphpad, LaJolla, CA, USA). Statistical comparisons between two-groups was performed using the unpaired Student’s *t*-test. All data are mean±SD represented by the error bars. Means were considered significantly different when p<0.05. P values were adjusted for the number of times each test was performed.

## Results

### THS exposure results in metabolic syndrome

**Fig 1:**
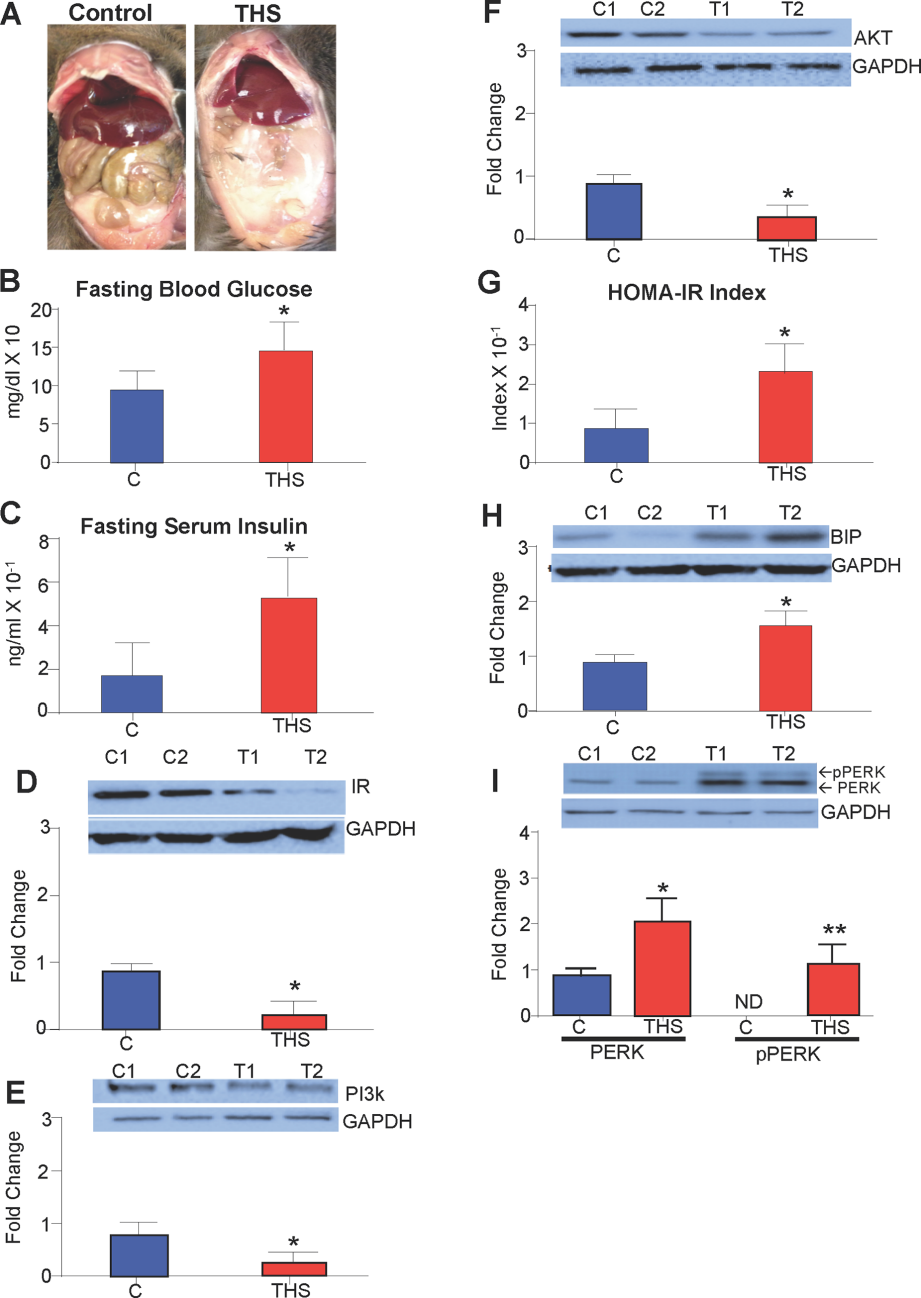
THS exposure results in metabolic syndrome. THS exposure results in visceral fat accumulation **(A)** increased fasting glucose levels **(B)** increasd Fasting insulin levels **(C)**. Western Blot analysis of the skeletal muscle shows reduced protein levels of IR **(D)**, PI3k **(E)** and AKT **(F)** in mice exposed to THS. **(G)** The HOMA-IR index (fasting blood glucose X Fasting insulin/22.5) of mice exposed to THS was higher than that of controls. **(H-I)** Increased protein levels of BIP, total PERK and p-PERK are observed in the THS exposed mice. *All data are Mean ± SD* * p< 0.05, ** p<0.01. n = 12. *P values were adjusted for the number of times each test was run*. *“Fold change” on western Blot graphs indicate fold change to control*.

THS-exposed mice have ectopic visceral fat accumulation (**Fig 1A**), which is a characteristic of metabolic syndrome and potential problems with insulin signaling because insulin regulates lipid metabolism and storage [19]. Therefore, we tested fasting blood glucose levels in the THS-exposed mice. Blood was consistently collected from the mice after a 12 hr fasting period in order to ensure that both glucose and insulin were at base levels. With this test we found that the fasting blood glucose levels in 49% of these mice were higher compared to controls (**Fig 1B**). The remaining 51% of the population of mice had blood glucose levels similar to controls. We concentrated our studies on the hyperglycemic mice to ensure that we understand the mechanisms by which THS exposure induces insulin resistance. Including the entire mouse population (ie. including the population with no response to THS)would artificially reduce the effects and not allow us to achieve significance for the susceptible population.

We asked whether the increase in circulating glucose in the susceptible population was due to insulin deficiency, hence we tested the levels of insulin in the blood of THS-exposed mice and found that fasting serum insulin levels were increased (**Fig 1C**). Glucose entry into muscle cells depends on a signal transduction cascade initiated by insulin interaction with the insulin receptor (IR), which results in activation of molecules inside the cells such as PI3K and AKT and facilitates glucose entry into the cells by GLUT 4 channel fusion with the plasma membrane [19]. Therefore, we tested for disruption of key proteins involved in the insulin signaling cascade. Using western blot analysis, we observed that THS-exposed mice have decreased levels of IR (**Fig 1D**), PI3K (**Fig 1E**), and AKT (**Fig 1F**). To quantify insulin resistance, we used the homeostatic insulin resistance index (HOMA-IR) [20–21] and found that mice exposed to THS have a significantly higher index than control (**Fig 1G**). Because we observed increased insulin production, we investigated the levels of endoplasmic reticulum (ER)stress in the pancreas. We evaluated the levels of two cellular stress biomarkers that are characteristic of ER stress [22,23], the binding immunoglobulin protein (BIP/HSPA5) and phosphorylated protein kinase RNA-like endoplasmic reticulum kinase (p-PERK); both BIP and pPERK are increased (**Fig 1H and 1I**).

### THS induces oxidative stress and molecular damage

**Fig 2:**
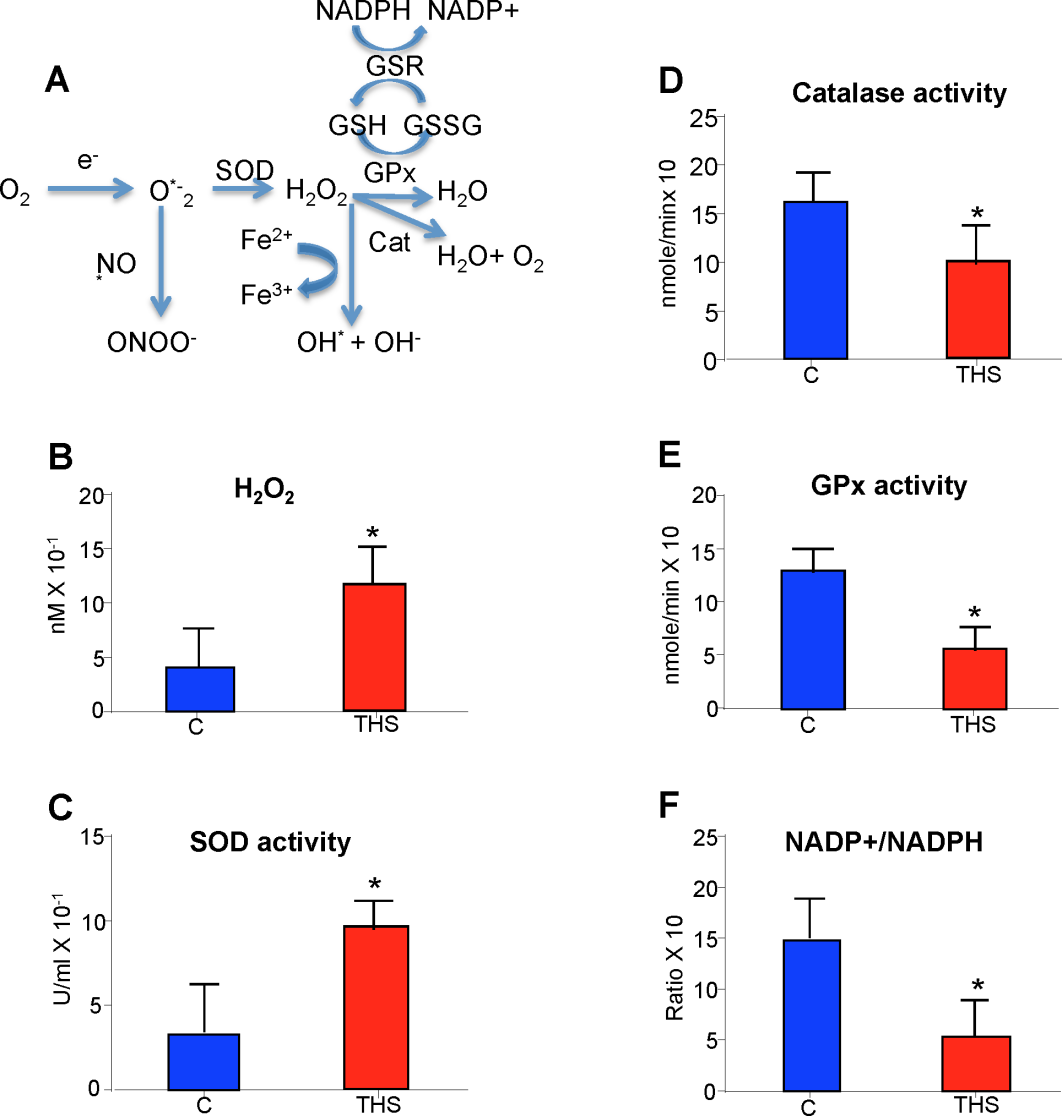
THS exposure results in oxidative stress in the skeletal muscle. **(A)** Schematic representation of oxidative stress response in cells. THS exposed mice have increased hydrogen peroxide **(B)** increased SOD enzymatic activity **(C)** decreased Catalse and GPx enzymatic activity **(D-E)** and the ratio of avaiblable NADP+ to NADPH is also decreased **(F)**. *All data are Mean ± SD* * = p< 0.05, ** = p<0.01. n = 12. *P values were adjusted for the number of times each test was run*.

Cigarette smoke toxins, such as those found in THS, are potent sources of reactive oxygen species (ROS) [24]. Upon injury, O_2_^.-^ anions are produced and then dismutated rapidly by superoxide dismustase (SOD) to produce hydrogen peroxide (H_2_O_2_) which is then broken down by the anti-oxidant enzymes catalase and glutathione peroxidase (GPx), to H_2_O and O_2_ (**Fig 2A)**. To determine whether mice exposed to THS have oxidative stress in skeletal muscle cells, we measure the levels of H_2_O_2_ and SOD activity. Both were increased in the skeletal muscle (**Fig 2B and 2C)**. However, H_2_O_2_ is a reactive molecule and is quickly broken down to H_2_O and O_2_ by catalase and GPx before it damages the cells. In THS-exposed mice, however, we see that the antioxidant potential of the muscle is severely reduced, as shown by the decrease in activity of Catalase and GPx (**Fig 2D and 2E)**. The antioxidant capability of GPx is derived from its reducing potential maintained by an adequate ratio of Glutathione (GSH) to Glutathione disulfide (GSSG). This ratio is maintained by the availability of electrons and hydrogen atoms supplied by NADPH in the cell. In the muscle of THS-exposed mice there is a low ratio of NADP+/NADPH (**Fig 2F**).

The increased levels of H_2_O_2_ can react wih Fe^2+^ ions in the tissue and undergo the Fenton reaction leading to production of hydroxyl radicals (**Fig 2A**). These, in turn, can lead to alterations in lipid peroxidation, protein modification, and formation of aducts in DNA, causing oxidative stress-mediated damage [25–28]. In the skeletal muscle of the THS-exposed mice we observed that lipid peroxidation (**S2A Fig**), protein nitrotyrosylation (**S2B Fig**), and DNA damage (**S2C Fig**) are all increased, altering their function and resulting in major physiological stress to the muscle.

### Antioxidant treatment decreases and “western diet” increases THS-induced damage

**Fig 3:**
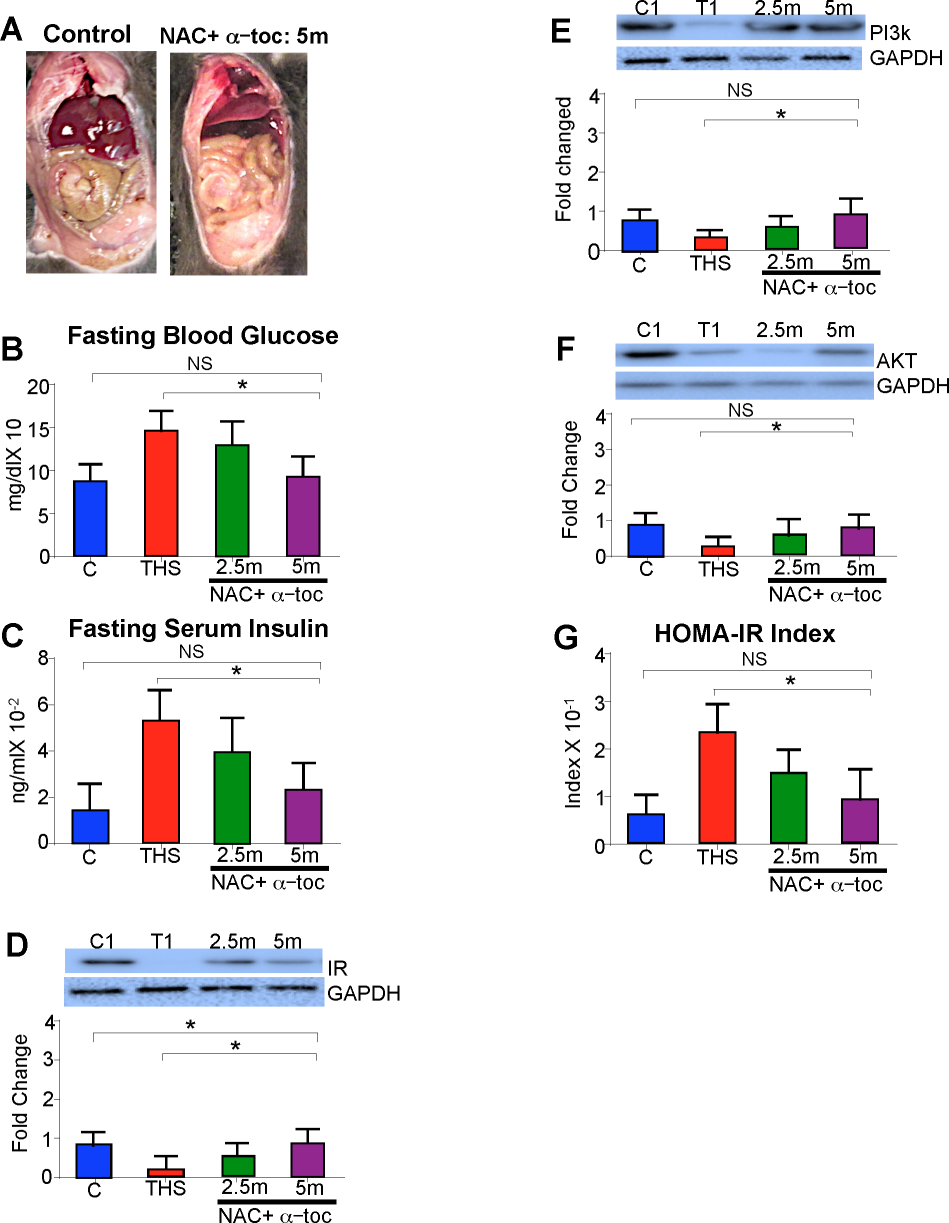
THS exposure in conjunction with antioxidant treatment for 2.5 and 5 months alleviates metabolic syndrome in a dose-dependent manner. **(A)** THS-exposed mice treated with antioxidants for 5 months have reduced visceral fat accumulation compared to THS exposed mice. Antioxidant+THS treated mice had reduced fasting blood glucose levels **(B)**, decreased fasting insulin levels **(C)**, greatly restored protein levels in insulin signaling proteins IR, PI3K and AKT **(D-F)**, and lower HOMA-IR index (fasting blood glucose X Fasting insulin/22.5) **(G)**. *All data are Mean ± SD* * = p< 0.05, ** = p<0.01. *NS = Not statistically significant*. n = 12. *P values were adjusted for the number of times each test was run*. *“Fold change” on western Blot graphs indicate fold change to control*.

We tested whether reversal of the THS-induced oxidative stress can reverse metabolic syndrome and cellular damage. To do this, we exposed the mice to THS for 5 months and then treated them intraperitoneally every other day for 2.5 months or 5 months with the antioxidants alpha-tocoperhol and N-acetylcysteine (NAC), while the mice still continued to be exposed to THS. alpha-toc inhibits lipid peroxidation and NAC stimulates the production of GPx [29–32]. We observed that 5 months of antioxidant treatment resulted in reduced visceral fat accumulation (**Fig 3A**), reduced fasting blood glucose (**Fig 3B**) and reduced fasting plasma insulin levels (**Fig 3C**). Antioxidant treatment also resulted in increased protein levels of IR (**Fig 3D**), PI3K (**Fig 3E**), and AKT (**Fig 3F**) in a dose-dependent manner. The HOMA-IR values for the antioxidant-treated mice also were reduced in a dose-dependent manner (**Fig 3G**).

**Fig 4:**
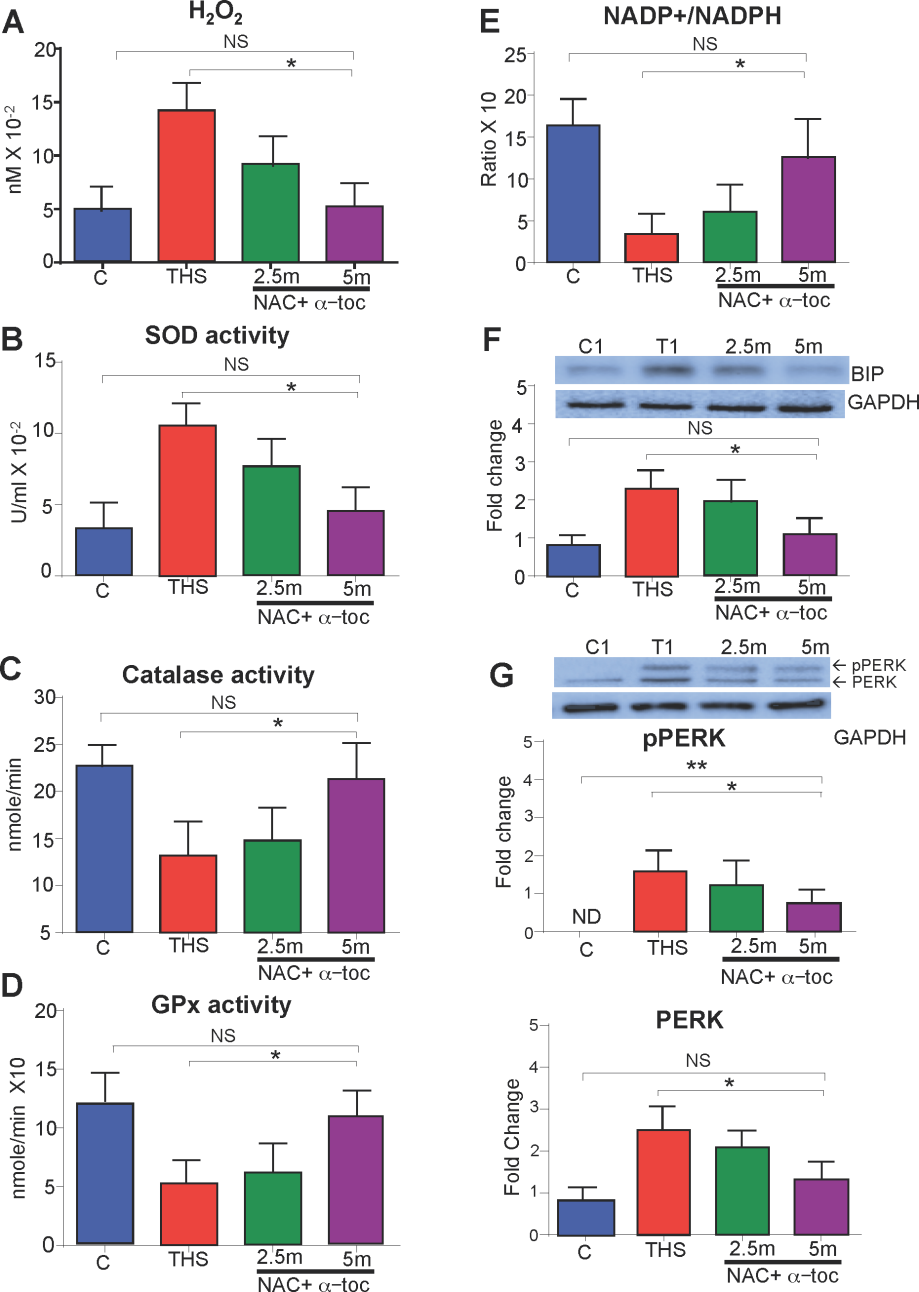
THS exposure in conjunction with antioxidant treatment 2.5 and 5 months reduces oxidative stress in a dose dependent manner. THS-exposed mice treated with the NAC+alpha-toc hαve progressively reduced levels of hydrogen peroxide **(A)**, progressively reduced SOD enzymatic activity **(B)**, progressively increased enzymatic acitivty of catalase and GPx **(C-D)**, progressively increased NADP+/NADPH ratio **(E)** and **(F-G)** progressivly less BIP, total PERK and pPERK protein levels. *All data are Mean ± SD*. * = p< 0.05, ** = p<0.01. *NS = Not statistically significant*. n = 6. *P values were adjusted for the number of times each test was run*. *“Fold change” on western Blot graphs indicate fold change to control*.

To determine whether antioxidant therapy reduced the THS-induced oxidative stress in the muscle, we again measured H_2_O_2_ levels and SOD, catalase and GPx activities. We observed that in the antioxidant-treated mice, H_2_O_2_ levels and SOD activity were significantly decreased (**Fig 4A and 4B**) and catalase and GPx activities were significantly increased (**Fig 4C and 4D**). We also observed a partial restoration of the reducing potential of the muscle as revealed by the increase in the NADP+/NADPH ratio (**Fig 4E**). The THS-induced increase in BiP, a protein upregulated during stress, was also reduced in the mice treated with antioxidants (**Fig 4F)**. With the reduction of insulin secretion observed in antioxidant-treated mice, we also saw reduction of both total PERK and p-PERK in THS-exposed mice treated with antioxidants, indicating that there was both improvement in the health of the pancreatic ß-cells as well as decreased ER stress and concomitant resolution of the UPR (**Fig 4G)**.

With the reduction of oxidative stress in the muscle when the mice were treated with the antioxidants, we determined whether these mice had reduced oxidative-mediated molecular damage compared to mice solely exposed to THS. THS-exposed mice treated with antioxidant for 5 months had reduced lipid peroxidation, reduced protein nitrosylation and reduced DNA damage. Indeed, at 5 months the levels were not significantly different from the control (**S3A–S3C Fig)**.

**Fig 5:**
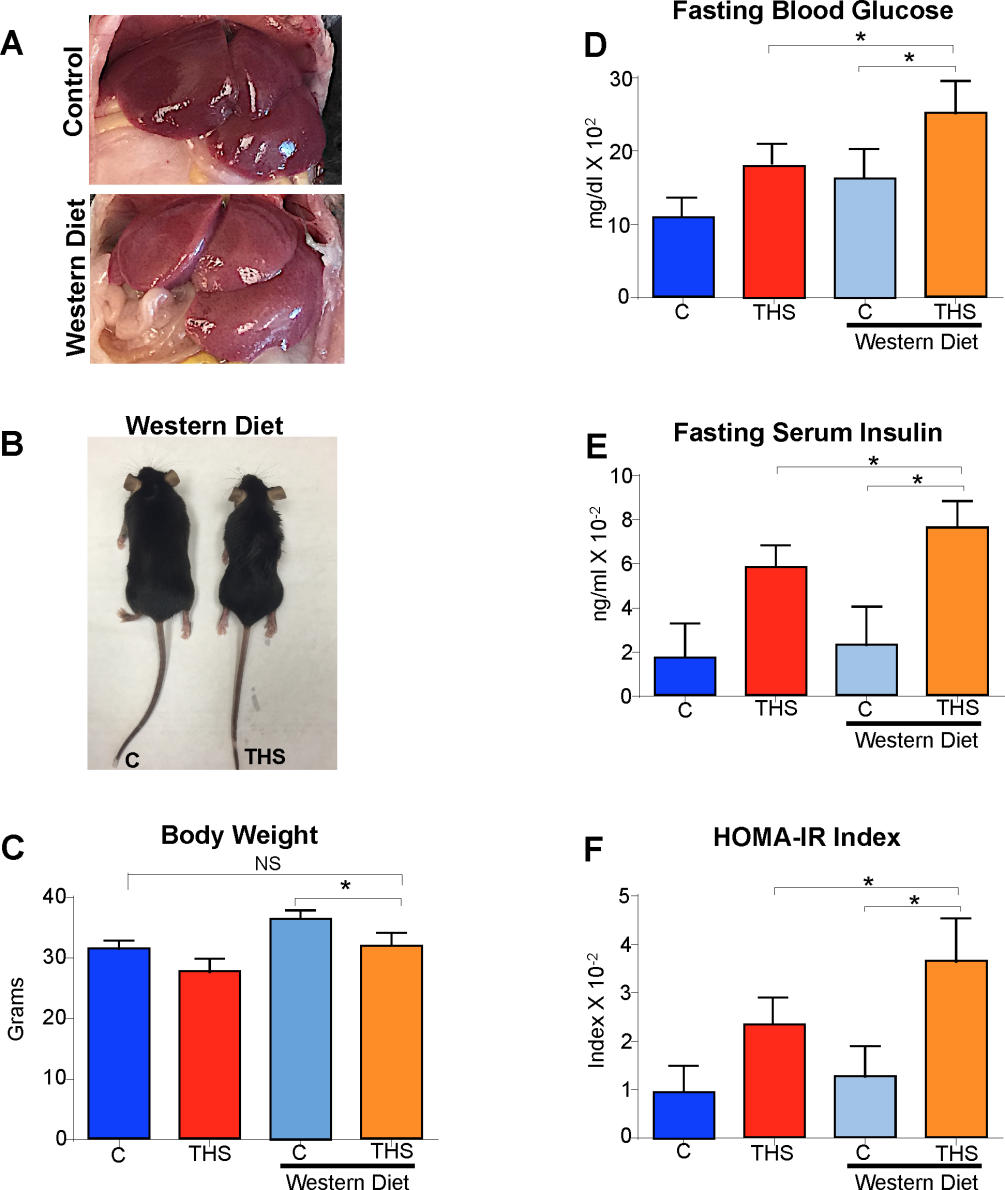
THS exposure in conjunction with “western diet” increases metabolic syndrome. THS exposed mice on “western diet” have increased fat accumulation in the liver **(A)**, are leaner than control mice on “western diet” (right mouse) **(B)**, weigh less than control mice on western diet **(C)**, have increased fasting blood glucose compared to control and THS-exposed mice **(D)**, have increased fasting insulin levels compared to control and THS-exposed mice **(E)**, and have an increased HOMA-IR index (fasting blood glucose X Fasting insulin/22.5) compared to control and THS-exposed mice **(F)**. *All data are Mean ± SD*. * = p< 0.05, ** = p<0.01. *NS = Not statistically significant*. n = 6. *P values were adjusted for the number of times each test was run*. *“Fold change” on western Blot graphs indicate fold change to control*.

To investigate the effect of increasing oxidative stress beyond that caused by THS on a standard diet, we placed mice exposed to THS on a “western diet” which is high in saturated fats and simple carbohydrates, and is a high source of reactive oxygen species [33–34]; (also see Methods). Mice were placed on the western or standard diet at weaning in conjunction with THS exposure for 5 months. In contrast to the THS-exposed mice on standard diet (which already show fatty liver) [16], those on “western diet” showed increased fat in the liver (**Fig 5A)**. Nevertheless, the THS-exposed mice on “western diet” were noticeably smaller (**Fig 5B)** and gained less weight than their control counterparts (**Fig 5C)** but still had significantly higher fasting blood glucose, serum insulin levels and high HOMA-IR index values compared to controls on the “western diet” alone or THS-exposed mice on a standard diet. (Fig **5D–5F****)**.

**Fig 6:**
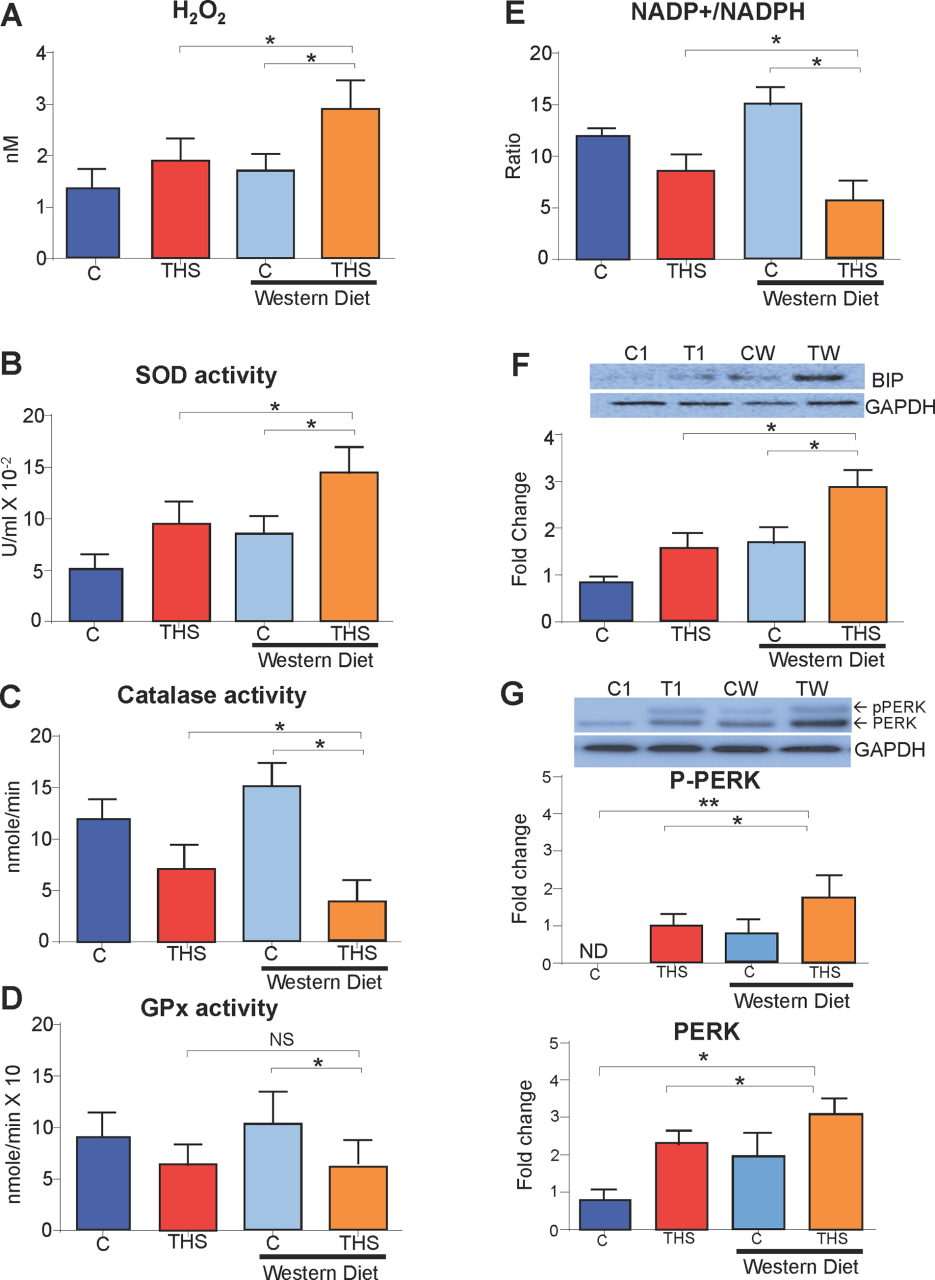
THS exposure in conjunction with western diet increases oxidative stress. THS-exposed mice on “western diet” have increased hydrogen peroxide compared to that of controls and THS-exposed mice **(A)**, have increased SOD enzymatic activity **(B)**, and have decreased catalase enzymatic activity **(C)**. However, GPx activity is not changed compared to control and THS-exposed mice **(D)**. In addition, THS-exposed mice on “western diet” have a decreased ratio of available NADP+ to NADPH compared to that of controls and THS-exposed mice **(E)**. THS-exposed mice on “western diet” have increased protein levels of BIP, total PERK and pPERK compared to THS-exposed mice, control mice and control mice on “western diet” (**F,G)**. *All data are Mean ± SD*. * = p< 0.05, ** = p<0.01. n = 6 * = p< 0.05, ** = p<0.01. *NS = Not statistically significant*. n = 6. *P values were adjusted for the number of times each test was run*. *“Fold change” on western Blot graphs indicate fold change to control*.

Lastly, mice exposed to THS for 5 months in conjunction with being fed a “western diet” had significantly increased H_2_O_2_ levels and SOD activity in the muscle (Fig **6A and 6B****)** and the antioxidant capacity of the skeletal muscle was further reduced (Fig **6C and 6D****)**. THS-exposed mice on the “western diet” have a decreased ratio of available NADP^+^ to NADPH in the the skeletal muscle compared to that of controls and THS-exposed mice on standard diet (**Fig 6E)**. Furthermore, compared to control mice, THS-exposed mice on the “western diet” had increased levels of BIP, total PERK and p-PERK (Fig **6F and 6G****)** and also had increased levels of lipid peroxidation, protein nitrosylation and DNA damage in the skeletal muscle (**S4A–S4C Fig)**.

## Discussion

**Fig 7:**
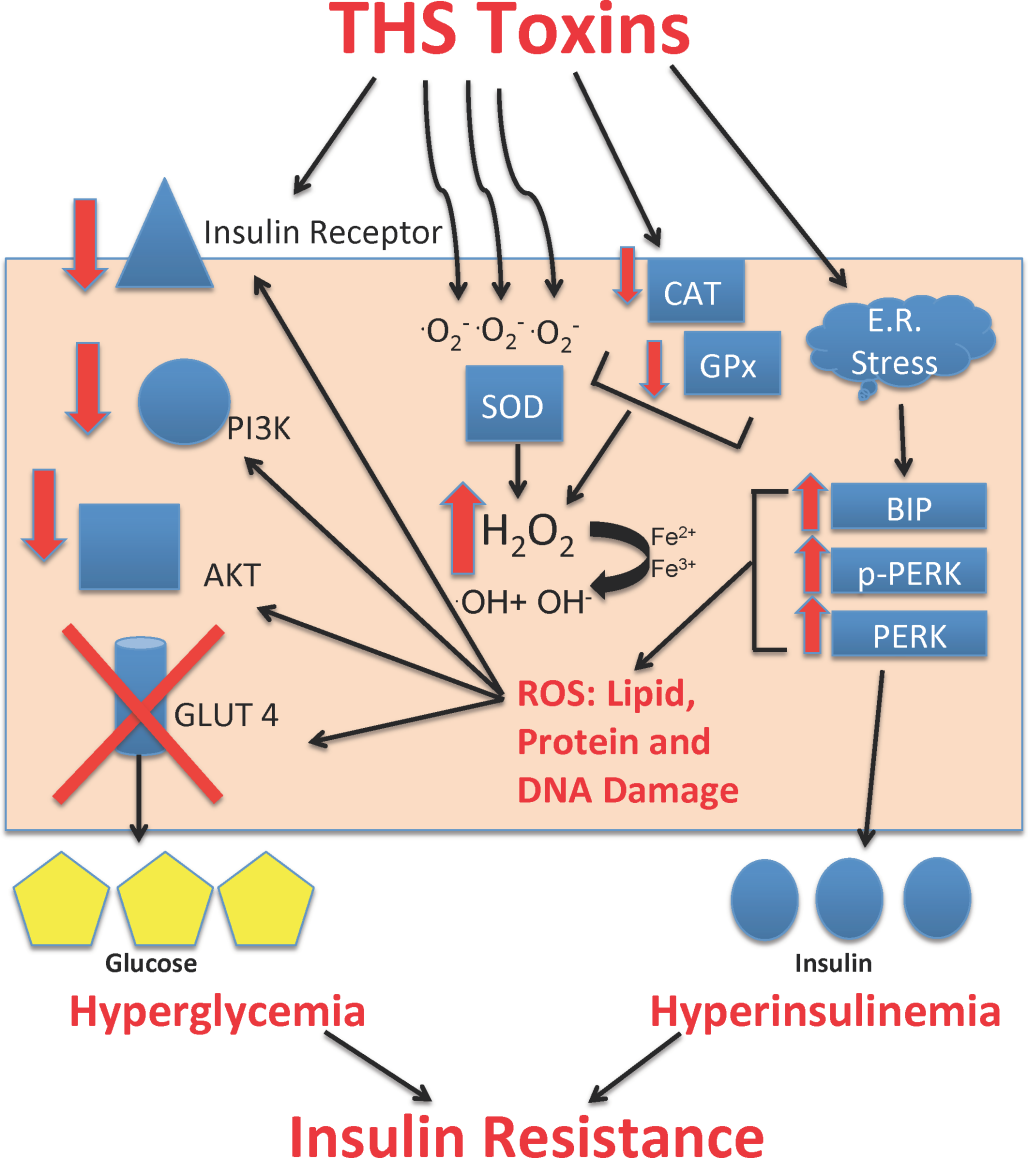
Working model of insulin resistance induced by oxidative stress as a result of THS exposure. THS toxins are a source of reactive oxygen species. These oxidants can decrease the expression and activity of insulin-signaling molecules such as IR and its downstream signaling molecules PI3k and, AKT ultimately leading to the inability of GLUT 4-containing vesicles to fuse with the plasma membrane. In response to excessive levels of the superoxide anion (O_2_^.-^), SOD is activated and dismutates O_2_^.-^ radicals into hydrogen peroxide. THS toxins, however, reduce the activity of catalase, preventing the conversion of H_2_O_2_ into water and O_2_. As hydrogen peroxide accumulates in the cell, it undergoes tertiary reactions with iron resulting in the formation of the OH radicals, which damages lipids, proteins, and DNA. THS toxins also lead to ER stress as BIP, PERK, and pPERK levels are elevated. Together, these effects lead to poor insulin signaling, leading to hyperglycemia and hyperinsulinemia, putting THS-exposed mice in a class of being insulin resistant.

We have shown that THS exposure leads to increased production of reactive oxygen species in the skeletal muscle of mice exposed to THS under conditions that mimic human exposure. This leads to a state of oxidative stress which, in turn, leads to cellular and molecular damage to proteins, lipids and DNA, affecting key insulin signaling molecules and as a result leading to hyperglycemia and hyperinsulinemia, a condition known as insulin resistance (**Fig 7**). In our model, we observe that about 49% of the population of exposed mice develop symptoms of insulin resistance and metabolic syndrome, with increases in fasting blood glucose, fasting serum insulin, and HOMA-IR index, leading to ectopic accumulation of fat in the viscera. Visceral fat accumulation is a hallmark of insulin resistance and metabolic syndrome; it is observed in 75% of humans with insulin resistance and type-2 diabetes. Furthermore, the HOMA-IR index, indicates that mice exposed to THS have a higher index of insulin resistance than the non-exposed mice. These findings mimic metabolic syndrome in humans in that not all people exposed to smoke develop signs of insulin resistance. In addition, although for this study we used only mice with high glucose levels, we can still see varying degrees of severity in insulin resistance, indicating that other factors such as genetic variability and susceptibility to disease also contribute to the variation.

The work presented here focuses on the skeletal muscle because it is the major peripheral tissue involved in glucose uptake and heavily dependent on insulin signaling to properly metabolize glucose [35]. Alterations to the skeletal muscle are frequently associated with the development of insulin resistance and type-2 diabetes in both mouse and human populations [35,36]. The data we obtained on the oxidative stress state of the skeletal muscle of THS-exposed mice shows that THS exposure leads to the accumulation of H_2_O_2_, which, although less reactive than superoxide anion, is still reactive and needs to be further broken down by catalase and GPx [37] to H_2_O and O_2_. In THS-exposed mice, however, we observe that the H_2_O_2_ produced in the cells is not broken down because catalase and GPx activities are low, resulting in accumulation of ROS in the skeletal muscle. One potential explanation as to why GPx activity is low is that the ratio of reduced to oxidized nicotinamide adenine dinucleotide phosphate (NADP+/NADPH) is low.

The elevated levels pPERK and BiP in the pancreas of THS-exposed mice indicate that THS induces ER stress in mice, with possible ER dysfunction, over the course of the exposure. THS toxins are a source of ROS as well as a cause of ROS formation in the skeletal muscle. pPERK has been shown to be elevated when insulin production increases [23], which correlates well with our finding that production of insulin is increased in THS-exposed mice. Increased BIP levels and phosphorilation of PERK are indicative of the unfolded protein response (UPR), suggesting that the pancreas is under stress in an attempt to manage the hyperglycemia.

This implies that THS-induced oxidative stress leads to cellular damage that then leads to the insulin-resistant state that we measured. This insulin-resistant state overburdens the pancreas to a point where a large output of insulin occurs leading to the UPR through increase in BIP and p-PERK. This constant activation of PERK can disrupt the normal levels of active PERK required not only for maintenance of UPR but also maintenance and regulation of the pancreatic ß-cells. The DNA damage resulting from THS exposure and the ability of THS to hypermethlyate DNA [38], coupled with this increase in PERK, can increase the possibility of genetic or epigenetic changes in genes involved in cell-cycle control or tumor suppression in the pancreatic ß-cells. These data lead us to speculate that constant exposure to THS toxins could prime the pancreas for eventual development of cancer later in life.

To reverse the THS-induced oxidative stress we used NAC and alpha-tocopherol, both commonly used antioxidants in humans and rodents, to reverse oxidative stress [29–32]. α-tocopherol (vitamin E) is an important lipid-soluble antioxidant. It performs its functions as antioxidant in the glutathione peroxidase pathway and it protects cell and mitochondrial membranes from oxidation by reacting with lipid radicals produced in the lipid peroxidation chain reaction [29–30]. alpha-Tocopherol removes the free radical intermediates and prevents the oxidation reaction from occurring and, because it is soluble in lipids, it protects them from oxidative damage. NAC, on the other hand, is a modified amino acid and a precursor of glutathione, the primary antioxidant enzyme in the cell [31]. NAC exhibits direct and indirect antioxidant properties. Its free thiol group is capable of interacting directly with the electrophilic groups of ROS. NAC exerts its indirect antioxidant effect because it is a GSH precursor. GSH is a tripeptide composed of glutamic acid, cysteine and glycine in which the sulfhydryl group of cysteine neutralizes nitric oxide, sulfur oxide, and other components of cigarette smoke and pollution [29–30]. Therefore, using these antioxidant agents, we were able to reduce the oxidative stress and, as a result, also reduce the damage to proteins, lipids and DNA.

In order to confirm that THS-induced oxidative stress is central in causing the metabolic state of the muscle, we increased oxidative stress by feeding the mice a diet high in fats and simple sugars, and low in protein, vitamin and fiber content, known as the “western diet” [33–34]. We found that THS-exposed mice on the “western diet” gained significantly less weight than the non-THS-exposed control mice on the “western diet”. This suggests that this model can be used to study why humans exposed to FHS and SHS lose weight. Nicotine, as well as other constituents of tobacco smoke, can affect the brain as well as hormone levels of ghrelin and leptin, thereby curbing appetite and leading to weight loss in smokers [39]. Interestingly, however, cigarette smoke toxins can lead to alterations of the metabolic hormone adiponectin in ways that lead to re-distribution of fat deposition in the body and can lead to visceral fat accumulation, which is a source of inflammation, and oxidative stress [40]. This is probably why our THS-exposed mice weigh less than control mice but develop insulin resistance. Nicotine leads to decreased appetite but also creates accumulation of visceral fat which then leads to increased oxidative stress and eventually to the metabolic syndrome.

Our findings show that the observed insulin resistance and metabolic syndrome in THS-exposed mice are not associated with obesity but rather with the oxidants and toxins present in THS. This leads to the physiological oxidative stress to the body seen in non-obese type 2 diabetes (NODII) of humans. NODII occurs in individuals who are of normal weight and have normal diets with no genetic predisposition to insulin resistance. The mechanisms by which this type of diabetes occurs are not known, except that NODII patients have a history of physiological and/or psychological stress [41–42]. NODII accounts for 15% of cases of type II diabetes in the world and is considered a silent killer because individuals with this condition, which normally “look healthy”, are not tested for this condition. This delay in diagnosis allows for the damaging effects of hyperglycemia to persist undetected, taking their toll on multiple organ systems [42].

### Conclusion

THS exposure compromises the activity of the antioxidant enzymes and induces a state of oxidative stress in the skeletal muscle. Accordingly, we observe the accumulation of ROS that undergo tertiary reactions creating by-products that damage lipids, proteins, and DNA. These multiple levels of cellular and molecular damage affect the health and ability of cells to undergo proper insulin signaling. As a result, we see hyperglycemia in mice exposed to THS. Alterations in insulin signaling as a result of increased oxidative stress also lead to abnormal lipid metabolism in the muscle, liver and abdominal cavity of THS-exposed mice. Our data elucidate the cellular and molecular mechanisms by which insulin resistance and metabolic syndrome can occur through a mechanism other than obesity, leading to non-obese insulin resistance. Also, our results lead us to speculate that long term exposure to THS toxins can cause constant insult and injury to the pancreas and set the stage for the eventual development of pancreatic cancer. Our findings have the potential for direct application to humans because tobacco toxins are often present in human habitats. If the findings presented here are disseminated widely, we believe it is potentially possible to decrease the number of people developing non-obese type 2 diabetes by educating people about the dangers of THS exposure as well as using antioxidant therapy to control oxidative stress. It is therefore important to inform the public and leaders of society to influence public policy towards controlling the exposure of nonsmokers, in particular infants, children, and the elderly, to THS toxins.

In addition, these studies could potentially also shed light on the mechanisms of non-obese type 2 diabetes, and provide potential therapeutic targets for individuals with this disease. In future studies, THS exposure could be a reproducible and quantifiable model to determine how tobacco toxins can cause alterations to insulin signaling and sensitivity which can lead to diabetes in non-obese individuals.

## Supporting Information

S1 Fig Schematic representation of our THS exposure system. (TIFF)

S2 Fig THS exposed mice have oxidative-stress mediated cellular damage. **(A)** THS exposed mice have increased lipid peroxidation in the muscle compared to control mice. **(B)** Increased protein nitration in the muscle of mice exposed to THS compared to controls. **(C)** DNA damage is increased in the muscle of THS exposed mice. *All data are Mean ± SD*. * = p< 0.05, ** = p<0.01. n = 6. *P values were adjusted for the number of times each test was run*.
(TIFF)

S3 Fig THS exposure in conjunction with antioxidant treatment for 2.5 and 5 months reduces oxidative mediated damage in the skeletal muscle in a dose dependent manner. NAC+alpha-toc treated mice simultaneously exposed to THS have **(A)** decreased lipid peroxidation compared to THS exposed mice. **(B)** decreased protein nitration compared to that of THS exposed mice. **(C)** decreased DNA damage compared to that of THS exposed mice. *All data are Mean ± SD* * = p< 0.05, ** = p<0.01. *NS = Not statistically significant*. n = 6. *P values were adjusted for the number of times each test was run*.
(TIFF)

S4 Fig THS exposure in conjunction with western diet increases oxidative-stress-mediated damage in skeletal muscle. THS exposed mice on western diet compared to control and THS-exposed mice have **(A) i**ncreased lipid peroxidation. **(B)** increased DNA damage **(C)** increased protein nitration and **(D)** decreased ratio of available NADP+ to NADPH. *All data are Mean ± SD*. * = p< 0.05, ** = p<0.01. *NS = Not statistically significant*. n = 6. *P values were adjusted for the number of times each test was run*.
(TIFF)

S1 Methods Detailed explanation of our experimental protocols and assays. (DOCX)
