# Correction: A Health Threat to Bystanders Living in the Homes of Smokers: How Smoke Toxins Deposited on Surfaces Can Cause Insulin Resistance

**DOI:** 10.1371/journal.pone.0153382

**Published:** 2016-04-06

**Authors:** Neema Adhami, Shelley R. Starck, Cristina Flores, Manuela Martins Green

There are errors in affiliation 2 for author Shelley R. Starck. Affiliation 2 should read: Department of Biochemistry and Biophysics/Howard Hughes Medical Institute, University of California San Francisco, San Francisco, California, United States of America
