# Correction: A Health Threat to Bystanders Living in the Homes of Smokers: How Smoke Toxins Deposited on Surfaces Can Cause Insulin Resistance

**DOI:** 10.1371/journal.pone.0208056

**Published:** 2018-11-20

**Authors:** Neema Adhami, Shelley R. Starck, Cristina Flores, Manuela Martins Green

There is an error in Fig 1, panel F. Please see the complete, correct Fig 1 here.

**Fig 1:**
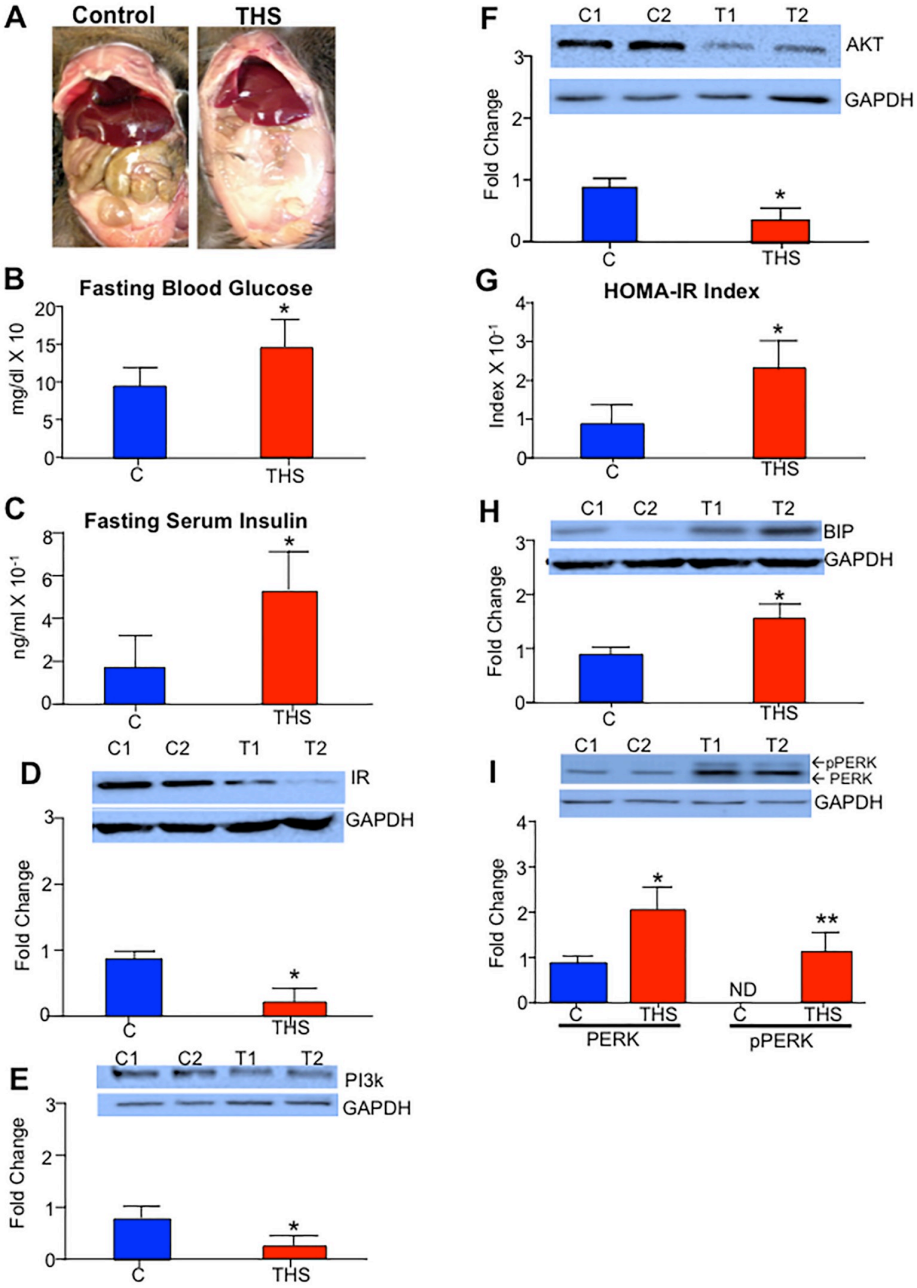
THS exposure results in metabolic syndrome. THS exposure results in visceral fat accumulation (A) increased fasting glucose levels (B) increasd Fasting insulin levels (C). Western Blot analysis of the skeletal muscle shows reduced protein levels of IR (D), PI3k (E) and AKT (F) in mice exposed to THS. (G) The HOMA-IR index (fasting blood glucose X Fasting insulin/22.5) of mice exposed to THS was higher than that of controls. (H-I) Increased protein levels of BIP, total PERK and p-PERK are observed in the THS exposed mice. All data are Mean ± SD * p< 0.05, ** p<0.01. n = 12. *P values were adjusted for the number of times each test was run*. *“Fold change” on western Blot graphs indicate fold change to control*.
