# Expression of Concern: A Health Threat to Bystanders Living in the Homes of Smokers: How Smoke Toxins Deposited on Surfaces Can Cause Insulin Resistance

**DOI:** 10.1371/journal.pone.0289810

**Published:** 2023-08-03

**Authors:** The PLOS ONE Editors

A representative of UC Riverside has contacted the *PLOS ONE* Editors to notify us of concerns related to this article [1–3] following an institutional investigation which reported the following concerns:

- In view of the difficulties operating and maintaining the smoking machine used in this study, the investigation concluded it is unlikely that cages and fabric could have been consistently and reproducibly treated as described in the article. It was raised that no records were found that indicated total particulate matter (TPM) was regularly measured and recorded during the time period in question and for the generation of the data presented in the article.
- In Figs 1F, 3D, 3E, 4G and 6F, the bands for the proteins of interest and the respective GAPDH loading control bands are not aligned, raising a question about whether these protein bands originate from the same western blotting membrane as the loading controls. The raw data were not provided.
- The GAPDH loading control panels in Fig 1F and Fig 3D appear identical in the original article; Fig 1F was subsequently corrected [2]. The raw data were not provided.

In an editorial review of the article the *PLOS ONE* Editors further noted that the bands in lanes 2–4 of the AKT panel in Fig 3F appear similar to the bands in lanes 1–3 of the BIP panel in Fig 1H.

The *PLOS ONE* Editors have followed up with the corresponding author, who stated that TPM measurements were accurately recorded during the study period and repairs made to the smoking machine were documented; however, records from the period in question are no longer available. The corresponding author stated that they had access to and reviewed the raw data prior to submission of the article and that the bands in lanes 2–4 of the AKT panel in Fig 3F are not the same as the bands in lanes 1–3 of the BIP panel in Fig 1H; however, the editors were unable to obtain the underlying blot images for the above-mentioned figures or the individual-level quantitative data underlying the accompanying charts in those figures. The editors were further unable to confirm which, if any, underlying data for this study remain available upon request at the time of preparation of this notice.

The *PLOS ONE* Editors issue this Expression of Concern to inform readers about the concerns that have been raised but could not be fully resolved in editorial follow up and that the underlying data for the study have not been made available in accordance with the published Data Availability statement.

PLOS has been informed that litigation was initiated by the corresponding author against UC Riverside and at the time of this notice’s publication the litigation remains pending.
