# Supplementary figures and images for: A Health Threat to Bystanders Living in the Homes of Smokers: How Smoke Toxins Deposited on Surfaces Can Cause Insulin Resistance
Source: PLoS One. 2016 Mar 2;11(3):e0149510. doi: 10.1371/journal.pone.0149510 (PMC4774920; doi:10.1371/journal.pone.0149510)

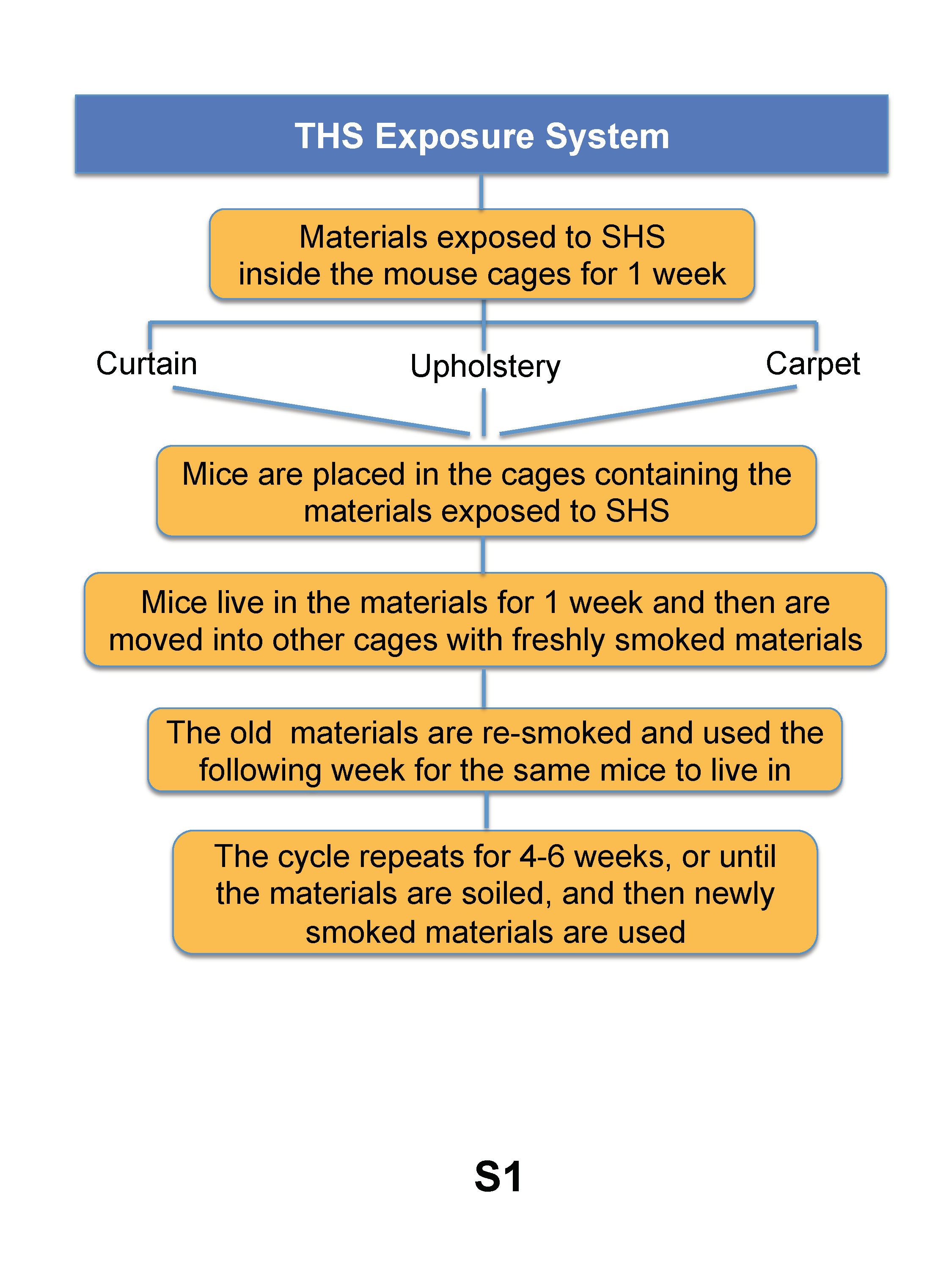

Supplement: S1 Fig — (TIFF) [file pone.0149510.s001.tiff]

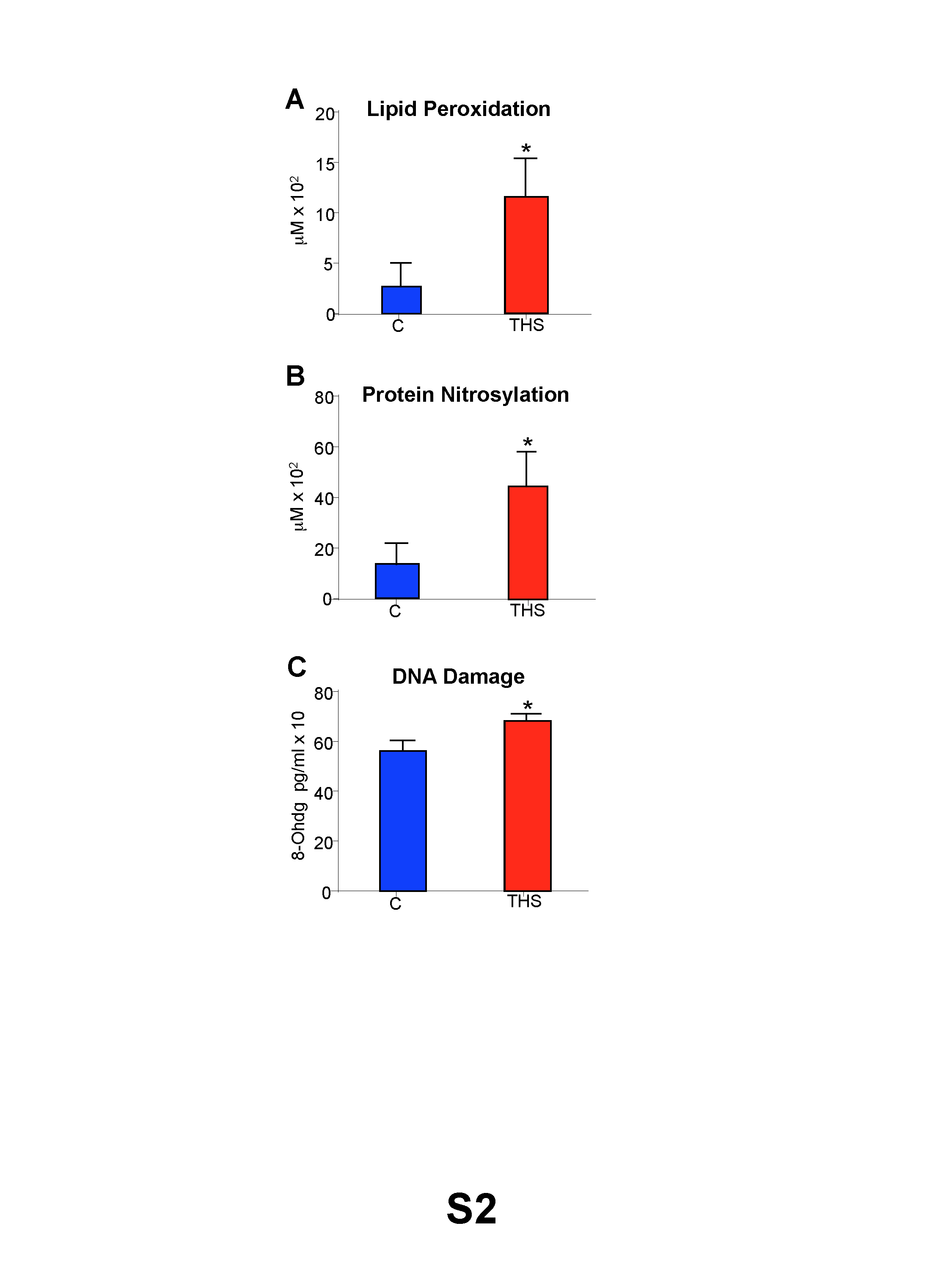

Supplement: S2 Fig — (A) THS exposed mice have increased lipid peroxidation in the muscle compared to control mice. (B) Increased protein nitration in the muscle of mice exposed to THS compared to controls. (C) DNA damage is increased in the muscle of THS exposed mice. All data are Mean ± SD. * = p< 0.05, ** = p<0.01. n = 6. P values were adjusted for the number of times each test was run. (TIFF) [file pone.0149510.s002.tiff]

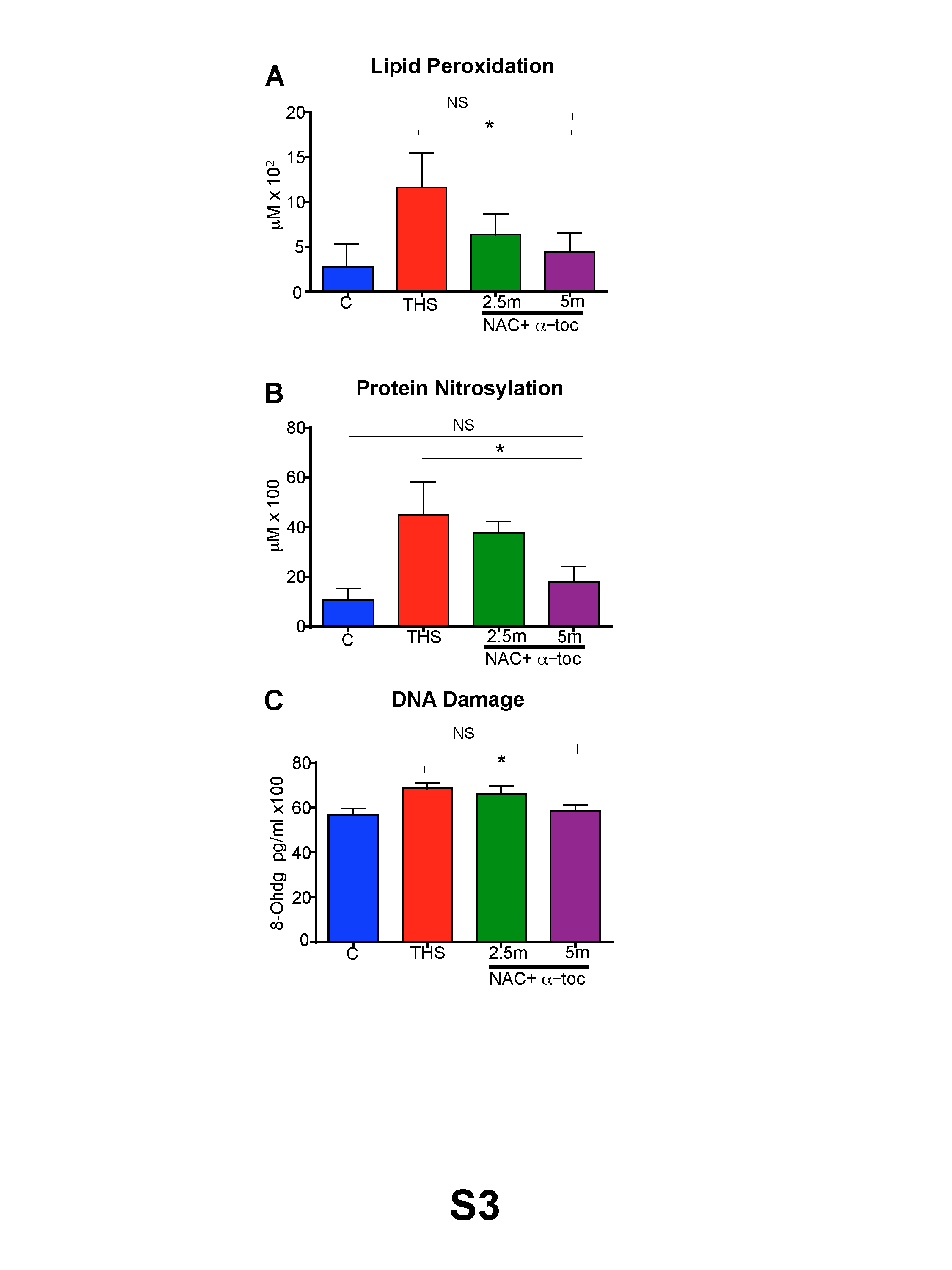

Supplement: S3 Fig — NAC+alpha-toc treated mice simultaneously exposed to THS have (A) decreased lipid peroxidation compared to THS exposed mice. (B) decreased protein nitration compared to that of THS exposed mice. (C) decreased DNA damage compared to that of THS exposed mice. All data are Mean ± SD * = p< 0.05, ** = p<0.01. NS = Not statistically significant. n = 6. P values were adjusted for the number of times each test was run. (TIFF) [file pone.0149510.s003.tiff]

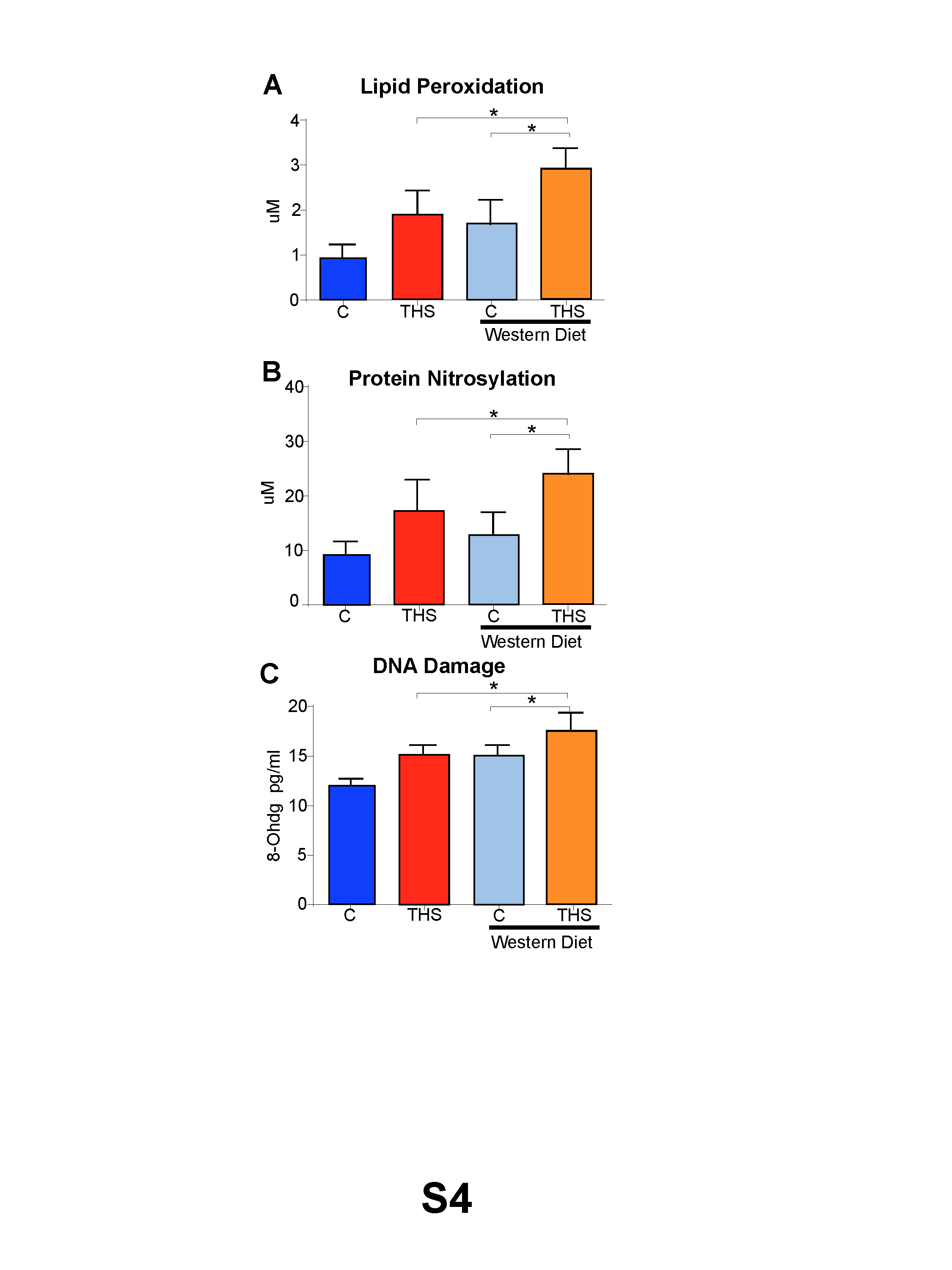

Supplement: S4 Fig — THS exposed mice on western diet compared to control and THS-exposed mice have (A) increased lipid peroxidation. (B) increased DNA damage (C) increased protein nitration and (D) decreased ratio of available NADP+ to NADPH. All data are Mean ± SD. * = p< 0.05, ** = p<0.01. NS = Not statistically significant. n = 6. P values were adjusted for the number of times each test was run. (TIFF) [file pone.0149510.s004.tiff]
