# Supplementary material for: A Health Threat to Bystanders Living in the Homes of Smokers: How Smoke Toxins Deposited on Surfaces Can Cause Insulin Resistance
Source: PLoS One. 2016 Mar 2;11(3):e0149510. doi: 10.1371/journal.pone.0149510 (PMC4774920; doi:10.1371/journal.pone.0149510)
Supplement: S1 Methods — (DOCX) [file pone.0149510.s005.docx]

**Materials and Methods**

**Western Blot Analysis:** We used procedure previously published by us (18). Briefly, skeletal muscle and pancreatic tissue were homogenized in RIPA lysis buffer containing protease and phosphatase inhibitors and the supernatants collected after 3X centrifugations at 4 ^o^C for 30 mins at 15,000rpm. Three rounds of centrifugation were required to remove the lipid that accumulates in the muscle of THS exposed mice that would affect the western blot results. Protein concentration of the supernatants was determined using the Bradford assay. 100µg of protein was loaded in 10% SDS-PAGE gels and ran at 50V through the stacking gel for 1hr and 90V through the separating gel for 2 hr. The proteins were transferred to nitrocellulose using a cold-wet transfer method for 1 hr at 90 volts, then washed with Tris-Buffered Saline and Tween 20 (TTBS) and blocked for 1 hr in 5% milk at RT. Membranes where incubated overnight at 4^o^C in a 1:5000 dilution of Insulin Receptor β Mouse mAb  ((L55B10; Cell signaling Technology Cat# 3020S); 1:1000 dilution of Akt Mouse mAb  (pan- 40D4; Cell signaling technology Cat#2920) ; 1:1000 dilution of Rabbit anti-BiP from Proteintech Cat.#11587-1-AP or Rabbit anti-PERK (C33E10)from Cell Signaling (Cat. #3192S) , in 5% Milk. After incubation, the membranes were washed with TTBS, incubated in HRP-conjugated anti-rabbit secondary antibody at a 1:5000 dilution in 1% milk for 1 hr at RT for all antiobidies and then developed using SuperSignal West Dura Extended Duration Substrate and the Bio-Rad Image gel developer and software.

**Hydrogen Peroxide Assay-** We used Caymans’s Chemical Hydrogen peroxide assay kit Cat# (7014200). 80μl of skeletal muscle homogenate or 80μL of each hydrogen peroxide standard were added to a 96 well plate. 10μL of assay buffer and 10μL was catalase solution were added to each well and the reaction was started, by adding 10μL of enzyme reaction solution, which was composed of Hydrogen Peroxide Detector ADHP and Horseradish Peroxidase. The plate was incubated for 30 min at RT and the fluorescent intensity of read (excitation = 530 nm; emission = 590 nm). Concentration of the samples was obtained from the standard curve.

**SOD activity assay:**We used Cayman’s Chemical SOD activity kit (Cat# 706002). 10μl of skeletal muscle homogenate or 10μL of each SOD standard were added to a 96 well plate. 200μl of Radical detector, which contained a tetrazolium salt solution, was added to each well. The reaction was initiated then with the addition of 20μL of xanthine oxidase. The plate was covered and shaken for 30 mins on a plate shaker at RT and optical density read at 440-460 nm. Concentration of samples were compared to standards to determine the activity of SOD based on the dismution of the superoxide radical, calculated from the absorbance’s and the times recorded upon the addition of xanthine oxidase.

**Catalase activity assay**- We used Cayman’s Chemical Catalase activity assay kit (Cat# 707002). 20μl of skeletal muscle homogenate or 20μL of each catalase standard were added to a 96 well plate. 100 μl of diluted Assay Buffer and 30 μl of methanol were added. The reaction was then initiated by adding 20 μl of diluted Hydrogen Peroxide. The plate was covered and incubated at RT for 20 mins. 30 μl of diluted Potassium Hydroxide was added to terminate the reaction along with 30 μl of Catalase Purpald as the chromogen. The plate incubated for 10 mins at RT. 10 μl of Catalase Potassium Periodate was added. The formaldehyde produced from these reactions was measured at absorbance’s of 540nm with 4-amino-3-hydrazino-5-mercapto-1,2,4-triazole (Purpald) as the chromogen. The time reaction of this color change was quantified as catalase activity in the sample compared to the standard curve.

**GPx activity assay**- We used Cayman’s Chemical GPx activity assay kit (Cat# 703102) 20μl of skeletal muscle homogenate was added to a 96 well plate. 100 μl of Assay Buffer and 50 μl of Co-Substrate Mixture were added to the plate. The Co-substrate mixture contained reconstituted in water, the lyophilized powder of NADPH, glutathione, and glutathione reductase. Three background wells were also designated which contained 100 μl of Assay Buffer and 50 μl of Co-Substrate Mixture. Three positive control wells were also designated which contained 100 μl of Assay Buffer, 50 μl of Co-Substrate Mixture, and 20 μl of diluted GPx (control). The reactions were initiated by adding 20 μl of Cumene Hydroperoxide to the plate. The plate was shaken for a 3 secs to ensure mixing. The optical density of the plate was measured at 340nm once every min for 5 mins. The activity of Gpx in the samples was then determined by first obtaining the change in absorbance of the samples over the 5 minuet period. The same was done with the background wells and the positive control wells. Gpx activity was then calculated by dividing the change in absorbance at 340 nm, calculated for each sample, by the NADPH extinction coefficient of 0.00373 μM-1*.

**NADP/NADPH ratio** – We used the NADP/NADP Kit from Biovision (Cat# K347 ). 20 mg of muscle tissue was washed in cold PBS, homogenized in NADP/NADPH extraction buffer in a microcentrifuge tube and centrifuged at 14000 rpm for 5 min and the supernatant isolated. To detect total NADP/NADPH, 50 μl of extracted supernatant from the samples was added into a 96-well plate. 100 μl of the NADP Cycling Mix, which consist of NADP Cycling Enzyme mix and NADP Cycling buffer mix, was added to each well. The plate was incubated at RT for 5 min to convert NADP to NADPH. 10 μl NADPH developer was added into each well and the reaction was allowed to develop for 2hrs. The optical density of the plate was read at OD450 nm. Concentration of NADP and NADPH of the samples was obtained from the standard curve.Once both NADP and NADPH values for each sample were calculated, then the NADP value was divided by the NADPH value to provide each sample with a ratio.

**DNA Damage** - We used the 8-hydroxy-2-deoxy Guanosine (8-OH-dG) ELISA kit from Cell BioLabs Inc (Cat# STA-320) to quantify 8-hydroxy-2-deoxy Guanosine (8-OH-dG) in ng/ml. Previously extracted DNA was dissolved in water at 1-5 mg/mL and converted into single stranded DNA by incubating the sample at 95oC for 5 min following by chilling on ice. DNA was then digested to nucleosides by incubating the denatured DNA with 5-20 units of nuclease P1 for 2 hrs at 37oC at final concentration of 20 mM Sodium Acetate, pH 5.2, followed by treatment of 5-10 units of alkaline phosphatase for 1 hr at 37oC in a final concentration of 100 mM Tris, pH 7.5. The reaction mixture was then centrifuged for 5 min at 6000 g and the supernatant was used for the 8- OHdG ELISA assay. 50 μL of experimental samples or 8-OHdG standards were added to the wells of the 8-OHdG Conjugate coated plate. Incubation was done at RT for 10 min on an orbital shaker. 50 μL of anti-8-OHdG antibody was added to each well, and the plate was incubated at RT for 1hr. The plate was then washed 3 times with Wash Buffer. After the last wash, 100 μL of secondary Antibody-Enzyme Conjugated was added to all the wells. Incubation was done at RT for 1 hr followed by 3 washes. 100 ul of Substrate Solution containing 3,3’, 5,5’Tetramethylbenzidine was added to each well and incubated at RT. The enzyme reaction was then stopped by addition of 100 μL of Stop Solution into each well. Optical density of the plate was read on a spectrophotometer at 450 nm and compared to the standard curve generated by the standards

**Nitrosylation of protein** - We used the OxiSelect Nitrotyrosine ELISA Kit from Cell Biolabs Inc (Cat#STA-305). 30 μl of skeletal muscle homogenate or nitrated BSA standard were added to a 96-well ELISA plate and incubated at RT for 10 min. Anti-nitrotyrosine antibody was then added to each well and the plate was incubated at RT for 1 hr. The plate was then washed 3 times with 1X Wash Buffer followed by an HRP-conjugated secondary antibody. Incubation was done at RT for 1 hr and then washed again with the 1X Wash Buffer. The Substrate for the enzyme (3,3’, 5,5’- Tetramethylbenzidine (TMB)) was added to each well, including the blank wells and incubated at RT for 2-30 min. The enzyme reaction was stopped by adding Stop Solution. Absorbance was read of each microwell on a spectrophotometer at 450 nm. The nitrosylation in the unknown samples was then determined by comparison with the predetermined standard curve.

**Lipid peroxidation-** We used the OxiSelect TBARS Assay Kit from Cell Biolabs (cat # STA-330). 30 μl of skeletal muscle homogenate or MDA standards were added to separate microcentrifuge tubes followed by SDS-containing lysis solution and incubated for 5 min at RT. Thiobarbituric Acid (TBA) reagent was then added to each tube, the tube was then closed and incubated at 95°C for 45-60 min, then cooled in an ice bath for 5 min followed by centrifugation at 3000 rpm for 15 min. The supernatant was removed from the samples for butanol extraction to remove hemoglobin and its derivatives. Samples were then vortexed vigorously for 1-2 min and centrifuged for 5 min at 10,000 g. The butanol fraction was then transferred to another tube for further measurement. 200μL of the MDA standards and samples were then added to a 96 well microplate. Absorbance’s were read at 532nm. The MDA in the unknown samples was determined by comparison with the predetermined MDA standard curve.
